# Vaginal lichen sclerosus: report of two cases

**Published:** 2017-09

**Authors:** J Xavier, P Vieira-Baptista, A Moreira, R Portugal, J Beires, V Tanos

**Affiliations:** Serviço de Ginecologia e Obstetrícia, Centro Hospitalar de São João, Porto, Portugal; Serviço de Ginecologia e Obstetricia, Centro Hospitalar Trás-os-Montes e Alto Douro, Vila Real, Portugal; Serviço de Anatomia Patológica, Centro Hospitalar de São João, Porto, Portugal

**Keywords:** vulvar dermatosis, lichen sclerosus, lichen planus, mucosal lichen sclerosus

## Abstract

**Background:**

Lichen sclerosus most commonly affects the genital area. Contrarily to lichen planus, the involvement of the oral or vaginal mucosa is rare. Only four cases of vaginal lichen sclerosus have been described in the literature.

**Case report:**

The authors report two cases of postmenopausal women with a history of vulvar pruritus and burning. Both presented with lesions of the vaginal mucosa compatible with lichen sclerosus, and genital prolapse. Vaginal biopsies confirmed the diagnosis. Initial treatment with topical clobetasol was effective in one of the patients, but in the other patient line therapy with pimecrolimus, triamcinolone, and retinoids was needed.

**Conclusion:**

Vaginal lichen sclerosus may be underdiagnosed and genital prolapse may favour the development of vaginal lesions.

## Introduction

Lichen sclerosus (LS) is a common chronic inflammatory skin disease which typically involves the vulva, but that rarely affects the vagina. This report adds two more such cases to the four previously published (Longinotti et al., 2005; Pérez- Lopez et al., 2013; Zendell et al., 2013).

### Case 1

A 61 year-old woman presented with a seven- month history of vulvar pruritus, burning and dysuria, for which she was being treated with vaginal estrogen cream, without relief. Symptoms worsened at night. Except for a history of surgery of correction of cystocele and urinary incontinence, her clinical history was unremarkable. Consent to publish was obtained from the patient.

Gynaecological examination showed a lichenified plaque in the right labium major, lichenification of the inner aspect of the labia majora, para-urethral erosions on the left side, hyperkeratosis on the outer third of the vagina and fissures in the perineal raphe.

**Figure 1:**
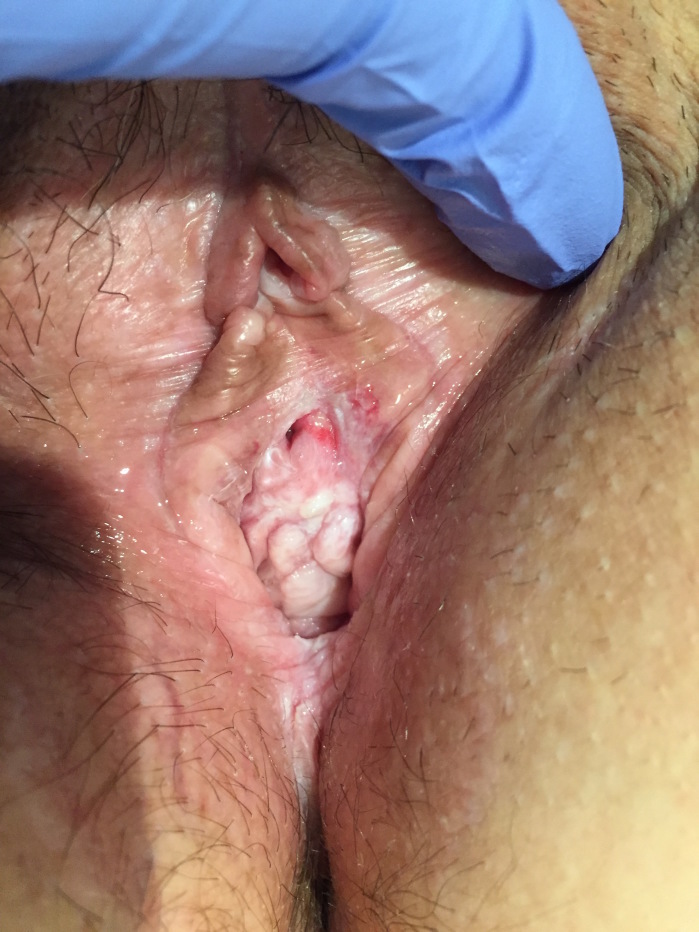
— Case 1: Vulvar and vaginal aspects prior to treatment (note the hyperkeratosis of the anterior wall of the vagina and the presence of vestibular erosions).

A cystocele grade I was also noticed (Fig. 1).

**Figure 2:**
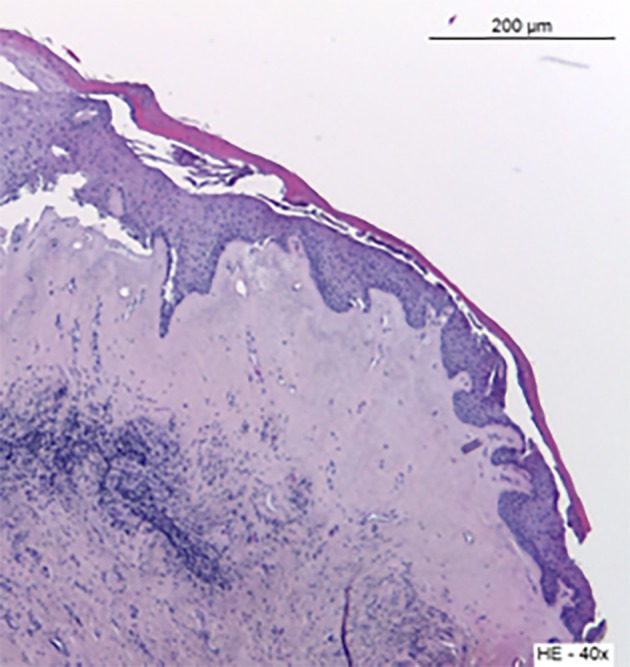
— Case 1: Histologial aspect of the involved vaginal mucosa, showing hyperkeratosis, subepithelial hyalinisation of collagen and deep lymphocitic inflammatory infiltrate (HE x40).

A biopsy of the anterior vaginal wall was performed. The histologic findings were consistent with the diagnosis of lichen sclerosus (Fig. 2).

The patient was prescribed topical clobetasol 0,05%, emollients and hydroxyzine. She was also recommended to keep applying vaginal estrogen cream twice a week.

According to the protocol of our institution, thyroid function was evaluated and found to be normal.

**Figure 3:**
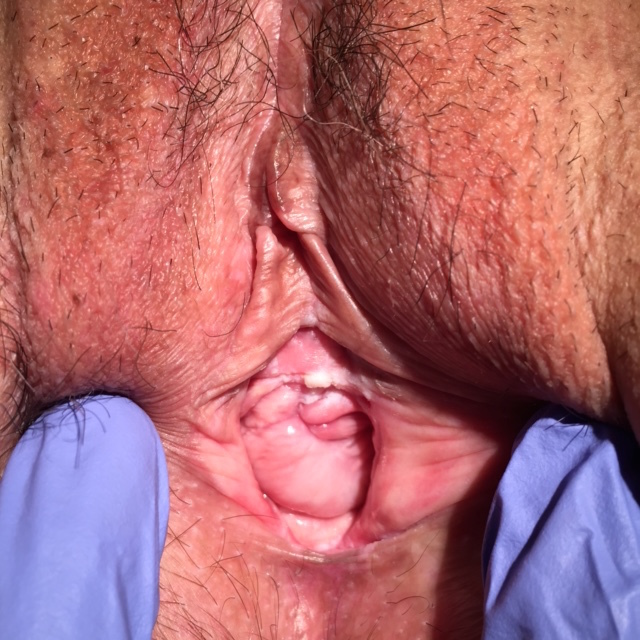
— Case 1: Improvement of the vulvar and vaginal aspects after treatment (2 months).

Two months later, she referred significant improvement of the symptoms and had significant remission of the vaginal hyperkeratosis (Fig. 3).

### Case 2

A 60-year-old woman was referred from another institution with a 3 years history of vulvar pruritus and burning. She had had a vaginal biopsy 2 years before, with an histological result of lichen sclerosus. She was using topical estriol, clobetasol and emollients, without a significant improvement. The patient had a history of hysterectomy with bilateral adnexectomy at the age of 52 years, for benign disease. Consent to publish was obtained from the patient.

**Figure 4:**
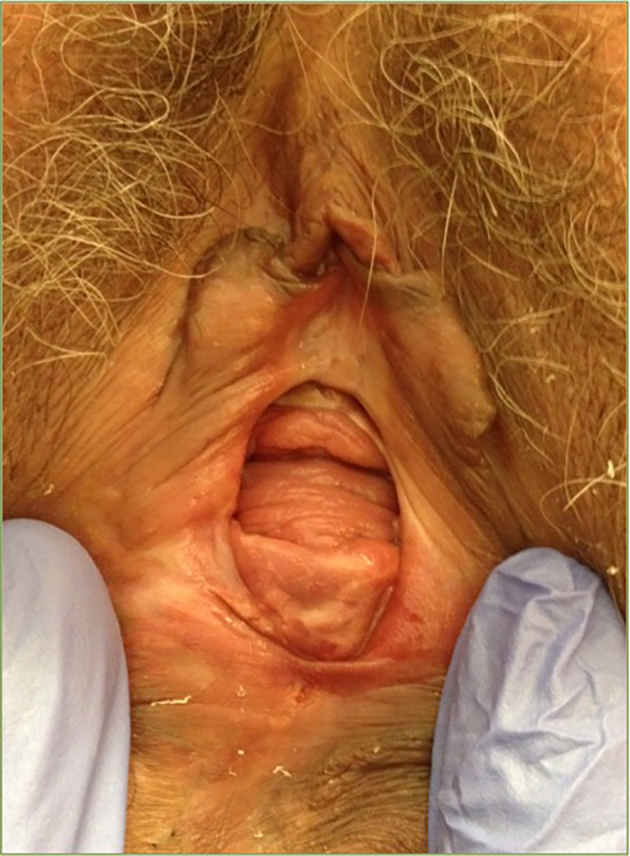
— Case 2: Vulvar and vaginal aspects prior to treatment.

Physical examination revealed a slight prolapse of the vaginal mucosa with lichenification of the posterior wall of the vagina (Fig. 4). The unexposed vaginal mucosa was normal. Vaginal wet mount was normal, except for a moderate inflammation. Analytically, thyroid function was normal.

The initial medication was stopped and the calcineurin inhibitor pimecrolimus 0.3% cream, twice a day, was started. As there was no improvement, clobetasol propionate and subcutaneous administration of triamcinolone was attempted, with some improvement of the symptoms. This time the wet mount revealed Doderlein lactobacilosis, which was treated with antibiotics.

One year after the initial appointment, the option was made to start treatment with retinoids and pregabaline (as she was also referring vulvar paraesthesias) with improvement of the symptoms and vulvovaginal lesions.

**Figure 5:**
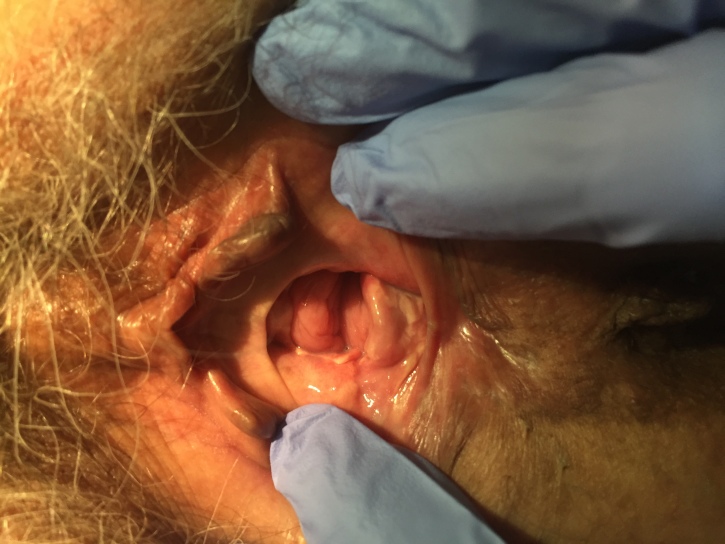
— Case 2: Improvement of the vulvar and vaginal aspects after treatment (2 years).

After two years of follow-up, the patient was asymptomatic and was discharged with the advice to visit her physician at least once a year (Fig. 5).

## Discussion

Lichen sclerosus is a chronic inflammatory skin disease that affects the anogenital area in 85-95% of cases (Longinotti et al., 2005; Bhargava and Lewis, 2013; Pérez-Lopez et al., 2013).

The diagnosis of LS is clinical and a biopsy is not always mandatory (Baptista et al., 2007). However, histological examination is needed if there is any suspicion of neoplastic change, treatment failure or if second-line therapy is needed (Neill et al., 2010)

LS rarely occurs with oral or vaginal involvement, unlike lichen planus (LP), that affects the vagina in about 70% of the cases (Pérez-Lopez et al., 2013; Sherlin et al., 2010).

We could only find four cases of vaginal lichen sclerosus in the literature (Longinotti et al., 2005; Pérez-Lopez et al., 2013; Zendell et al., 2013).

Vaginal lichen sclerosus may be underdiagnosed and underreported, since there have been several reports of involvement of other mucosae, such as the oral and urethral ones (Sherlin et al., 2010).

On the other hand, pruritus is not a symptom of the vaginal mucosa but a symptom of the skin, so these lesions will only be detected if associated with symptomatic vulvar lichen sclerosus. In these cases, a vaginal biopsy is important to confirm the diagnosis.

Both patients had favourable response to treatment, recommended for vulvar lichen sclerosus (topical corticoids, calcineurin inhibitors and retinoids).

Topical corticosteroids are the first line treatment, while calcineurin inhibitors (pimecrolimus or tacrolimus) are reserved for cases of failure or intolerance to those. The later are usually used twice a day, until improvement is achieved.

In both of our 2 cases and in 3 of the other 4 published, vaginal LS presented in a prolapsed mucosa. The exposure of the vaginal mucosa likely leads to chronic irritation and inflammation and, consequently to keratinisation. The keratinisation of the vaginal mucosa is likely to be the basis of the development of lichen sclerosus in this anatomical region (Pérez-Lopez et al., 2013; Zendell et al., 2013).

Vulvar lichen sclerosus, has an estimated malignancy rate of 2 to 6% (Micheletti et al., 2016). The risk of malignancy associated with vaginal LS is unknown, close follow-up is recommended.

Despite the lack of definite evidence, most specialist in the field believe that treating asymptomatic LS leads to a decrease in the risk of development of differentiated VIN and cancer. For the same principle, we suggest that the same attitude should be used in vaginal lichen sclerosus.

In conclusion, vaginal lichen sclerosus may be underdiagnosed and the genital prolapse seems to favour the development of vaginal lesions, due to changes (keratinisation) in the epithelium of vaginal mucosa.

## Conflict of interest

The authors have declared they have no conflicts of interest.
